# Testis Size Variation and Its Environmental Correlates in Andrew’s Toad (*Bufo andrewsi*)

**DOI:** 10.3390/ani12213011

**Published:** 2022-11-02

**Authors:** Ying Jiang, Li Zhao, Xiaofeng Luan, Wenbo Liao

**Affiliations:** 1 School of Ecology and Nature Conservation, Beijing Forestry University, Beijing 100083, China; 2 Key Laboratory of Southwest China Wildlife Resources Conservation (Ministry of Education), China West Normal University, Nanchong 637009, China; 3 Key Laboratory of Artificial Propagation and Utilization in Anurans of Nanchong City, China West Normal University, Nanchong 637009, China

**Keywords:** reproductive investments, environmental conditions, intraspecific variation, body condition

## Abstract

**Simple Summary:**

Life-history theory includes that trade-offs between reproduction and survival are critical for organisms to adapt to different environments. Therefore, understanding how organisms adapt their reproductive investment can provide insights into the evolution of life history. Our results showed that the testes size of *Bufo andrewsi* significantly differed among populations. We found no geographic trends explaining the variability in testes size, as relative testes size did not vary with altitude and/or latitude. Although rainfall cannot directly affect testes size, the coefficient of variation of temperature showed effects on testes size, indicating declined male reproductive investment under environments with fluctuating temperature and thus highlighting important implications for amphibian conservation.

**Abstract:**

Reproductive investments influenced by environmental conditions vary extensively among geographically distinct populations. However, investigations of patterns of intraspecific variation in male reproductive investments and the mechanisms shaping this variation in anurans remain scarce. Here, we focused on the variation in testis size in 14 populations of the Andrew’s toad *Bufo andrewsi*, a species with weak dispersal ability but wide distribution in southwestern China, to establish whether male reproductive investment varies on an environmental gradient. Our analysis revealed a significant variation in relative testis size across populations, and a positive correlation between testis size and body condition. We, however, found no geographic trends explaining the variability in the testis size. The relative testis size did not increase with increasing latitude or altitude. We also found no relationship between relative testis size and rainfall, but a negative correlation with the coefficient of variation of temperature, with larger testes under stable environments. These findings suggest that the decreased male reproductive investment of this species may be a consequence of harsher or fluctuating environmental conditions.

## 1. Introduction

Life-history theory predicts that trade-offs between current reproductive investments and future reproductive success and/or survival enable an organism to cope with environmental changes [1,2,3,4,5,6,7]. This inevitable trade-off is reflected by testis size, a dominant measure of reproductive investment in males, varying extensively within and between species [8,9,10,11]. Much of the interspecific variation in testis size has been attributed to differences in sexual selection pressures [10,12,13,14]. For example, comparative studies on variation in relative testis size support the broad theoretical prediction that species under strong sexual selection should invest proportionately more in testicular structure [15,16,17].

Although sexual selection theory has successfully explained interspecific diversity in testes, the ecological factors driving interspecific variation in testes remain ambiguous. Nevertheless, there is increasing evidence that differences in environmental conditions among populations of the same species influence the intensity of sexual selection, thereby promoting local adaptation of the relevant reproductive traits [18,19,20,21,22,23,24,25]. For instance, testis size is positively correlated with latitude in several North American bird species [8] because greater sexual selection pressure has been reported at higher latitudes [26,27]. Moreover, many studies have demonstrated that abiotic variables such as temperature and rainfall can influence activity periods, thereby limiting the time for development, resource acquisition, and breeding activity [21,22,28,29,30,31,32], and ultimately affecting the strength of sexual selection and patterns of reproductive investment [18,28,33,34,35,36]. Consequently, species that inhabited harsh and unpredictable environmental conditions (e.g., low temperature and/or high altitudes) with shorter activity periods were assumed to decrease reproductive investment. However, studies investigating the large-scale environmental effects on the variation of testes have yielded mixed results [3,14,22,37,38,39,40,41,42].

Among the intraspecific studies investigating testis size along ecological gradients, testis size has been shown to decrease at higher latitudes in the common frog *Rana temporaria* [3], possibly because of decreased resource acquisition or weaker intrasexual competition (less male-biased operational sex ratio, OSR) [3,21,43]. An altitudinal decline of testis size in the plateau brown frog *R. kukunoris* [40] and Tibetan toad *Scutiger boulengeri* [44] can be explained by similar reasons. In another study, testes from the high-altitude population of the spot-legged treefrog *Polypedates megacephalus* are relatively larger than those from the low-altitude population, due to greater sexual selection pressure (male-biased OSR) at high-altitude populations [14]. Hence, the potential drivers of intraspecific variation in testis size remain poorly understood, and further detailed and large-scale studies are needed.

The Andrew’s toad (*Bufo andrewsi*), as an ideal study species, is widely distributed in subtropical forests of the Hengduan Mountains in China, at elevations ranging from approximately 750 to 3500 m [30,45,46]. Previous work on *B. andrewsi* has indicated that the high-altitude population attains longer longevity, larger body size, and larger relative organ size than the low-altitude population [30,47,48,49,50,51]. This is possibly due to exposure to harsher environmental conditions (lower temperature or less rainfall, reduced food availability, and shorter growing season) [52]. However, little is known about the environmental factors driving the intraspecific variation in the testis size of this species.

Here, we explored the effects of environmental conditions (i.e., latitude, altitude, temperature, and rainfall variables) on the intraspecific variation of testis size in *B. andrewsi*. Specifically, our aim was to (1) test whether testis size differs predictably among 14 populations, (2) further investigate whether testis size varies along a geographic gradient, (3) examine the effects of temperature and rainfall on the testis size of *B. andrewsi*. We expected that populations inhabiting areas with higher latitudes and/or altitudes, lower temperatures, and less rainfall would demonstrate a declined investment in testes.

## 2. Materials and Methods

### 2.1. Data Collection

**Figure 1:**
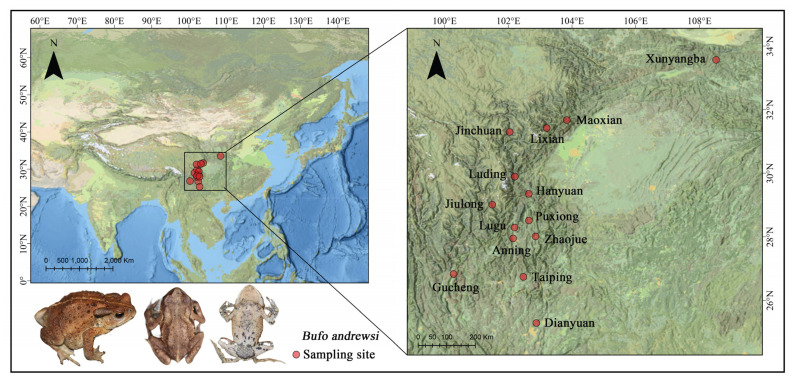
Geographic distribution of the sampled populations for *B. andrewsi*.

Between 2018 and 2019, we collected 331 sexually mature males from 14 populations ranging in altitude from 864 to 2367 m and spanning an 8° latitude in breeding seasons (Figure 1). For each population, we sampled 19–30 (mean ± s. e. = 23.6 ± 3.8) males at a single location between the end of March and the beginning of April in southern and western China with known longitude, latitude, and altitude (Table 1). All of the individuals were captured using a 12 V flashlight at night, and their sex was confirmed using their secondary sexual traits (e.g., nuptial pads in males and eggs in females). After being kept at room temperature for around 12 to 24 h, they were placed in a tank (1 × 0.5 × 0.8 m, L × W × H) containing fresh water [50,53]. Upon transfer to the laboratory, we sacrificed the individuals by single pithing [51,54], and then measured their snout–vent length (SVL) with a digital caliper to 0.01 mm and body mass with an electronic balance to 0.1 mg [55]. We preserved them in 4% phosphate-buffered formalin for tissue fixation [49,56,57]. All dissections and measurements were performed by Zhao Li. The study was conducted according to the guidelines of the Declaration of Sichuan and approved by the Ethics Committee of China West Normal University (2022008). All the methods of capturing and handling animals used in this study were approved by the Institutional Animal Care and Use Committee (IACUC) at China West Normal University (2022008).

After a maximum of nineteen months of preservation, the right and left testes were dissected and weighed separately to the nearest 0.1 mg with an electronic balance. Testis size was defined as the sum of the weights of the two testes. All measurements were taken blindly by identifying specimens by ID number without knowledge of the individuals’ identity. Previous studies have shown that the length of preservation time has no significant effect on the testis size of frogs [31,58].

### 2.2. Statistical Analyses

All statistical analyses were performed using R software 4.2.0 [59]. Continuous variables were log_10_-transformed to ensure they complied with the assumptions of parametric tests. Body condition was then calculated as the quotient of log_10_ (body mass) and log_10_ (SVL) to avoid collinearity, and we used it as one variable in the following analysis [60]. We used the R package ‘lme4′ to fit all models [61].

To test whether testis size differed significantly among populations, we performed one-way analyses of covariance (ANCOVA) in which population was entered as the fixed factor and testis size as the dependent variable. Body condition was added as a covariate to control for potential allometric relationships between body condition and testis size.

To examine whether geographical gradients (latitude and altitude) associated with the length of the breeding season and resource availability of *B. andrewsi* could explain variations in testis size among populations [48], linear mixed models (LMMs) were performed with the population as a random effect and body condition as a covariate. Furthermore, general linear models (GLMs) were conducted to assess the effect of geographical gradients on the average size of testis at the population level.

Next, we used linear mixed models (LMMs) to examine the impact of climate factors on variation in testis size at the individual level. Climate factors that have been shown to have a large impact on amphibian growth and development (i.e., mean annual temperature, annual rainfall, coefficient of variation (CV = SD/mean) of temperature, coefficient of variation (CV = SD/mean) of rainfall, the temperature during the breeding season, rainfall during the breeding season) [30,62] were assigned as fixed factors and the population was added as a random effect. We then analyzed the effect of climate factors on the average size of testes at the population level using general linear models (GLMs). Body condition was added as a covariate in all analyses.

## 3. Results

**Figure 2:**
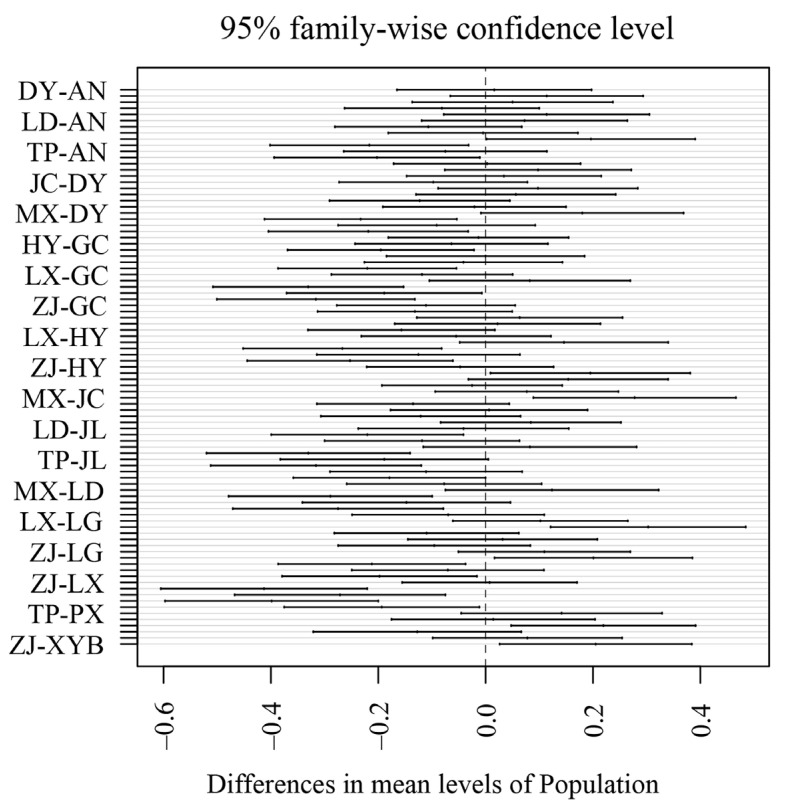
Results of post-hoc tests. Confidence intervals containing zero indicate that the difference in testis size between the two populations is not significant.

Testis size differed significantly among populations (ANCOVA; *F*_13,330_ = 14.260, *p* < 0.001, Table 2), and body condition was a significant covariate (*F*_1330_ = 181.950, *p* < 0.001, Table 2). Post-hoc analysis further showed that individuals from Maoxian displayed significantly different testes from other populations (Figure 2). Linear mixed models revealed that geographical variation in relative testis size can be explained by body condition (*t* = 13.564, *p* < 0.001, Table 2), but not by latitude (t = −0.378, *p* = 0.713, Table 3) and altitude (*t* = −0.271, *p* = 0.792, Table 3) at the level of individuals. When we further examined altitudinal and/or latitudinal variation in testis size at the population level, we found no convincing evidence of geographical trends in the relative testis size (all *p* > 0.05, Table 4).

To test the effects of climate factors on the relative size of testes, we first performed linear mixed models (LMMs) controlling for population (random effect). The LMMs revealed that although the relative testis size was not affected by rainfall (all *p* > 0.05, Table 3), it was significantly affected by the CV of temperature (*t* = −2.672, *p* = 0.021, Table 3). Males from stable environments with a lower CV of temperature had proportionally larger testes than those from unstable environments. Similar to the results of geographical gradients, GLMs revealed that the average testis size of each population was not correlated with climate factors when controlling the effect of body condition (all *p* > 0.05, Table 4).

## 4. Discussion

This study focused on the question of how environmental conditions affect variations of testis size in *B. andrewsi*. Our findings demonstrated considerable intraspecific variation in relative testis size among 14 populations. We also found a positive correlation between testes size and body condition across all individuals. However, inconsistent with our prediction, we found that the relative testis size does not decrease with increasing altitude and/or latitude, either at individual or population levels. Moreover, we surprisingly did not find trends for testis size to vary along a rainfall gradient. In the investigation of the CV in temperature, we found that relative testes size is negatively correlated with the CV in temperature. This finding is consistent with the prediction that stable environments favor larger testes, possibly implying that declined reproductive investment seems to be a general response to the fluctuating temperature environments in which the individuals have shorter activity periods. In the following, we discuss our findings associated with what was previously known from intraspecific studies on testis size variation.

Previous studies have suggested differences in male reproductive investment within and among populations are common throughout the animal kingdom (e.g., primates [63], frogs [3,21,22], and birds [64]). Indeed, our findings indicated that variation in relative testis size was significant among 14 populations. Breeding systems can influence male reproductive investment [3,65,66], with males having larger testes when competition for mates is high [67]. However, the species exhibits a male clasping a female in breeding ponds [48], and hence relative testis size was not correlated with the breeding system.

Interestingly, the positive correlation between body condition and relative testes size suggested that the condition-dependent testis size was observed in *B. andrewsi*. Indeed, previous studies on the positive allometric relationship between testis size and body condition have shown that relatively heavier males tend to exhibit disproportionately larger testes than lighter males in the animal kingdom [21,22,68,69,70,71,72,73,74,75,76,77]. It is well known that sperm production and the process of sperm competition can be energetically costly [34,40,43]. As a result, males with superior body condition may have more energy available to allocate to testes, thereby possibly increasing the chances of competition success and consequently gaining evolutionary benefits [34,65,78]. Other studies on frogs in China have made similar observations [23,34,40,44,73,79,80,81].

Geographical conditions have been suggested to influence male reproductive investments in frogs. Specifically, male frogs would decrease reproductive investments with increasing latitudes and/or altitudes, due to an increasingly short breeding season, declining levels of male–male competition for mates, or more limited resources to invest in reproduction [3,21,22,40,43,82], as has been evidenced in some frogs [3,21,40]. However, a study on the Asian grass frog *Fejervarya limnocharis* has found that high-altitude populations have larger testes, which may be attributed to environmental conditions affecting reproductive investments being mediated by differences in the availability of both resources and sexual partners [22]. In this study, we did not find any relationships between testis size and latitudes and altitudes across 14 populations, indicating that the extended re-acquisition of resources necessary for survival at high latitudes or altitudes cannot reduce the energy allocated to testis size. Similar results were reported in the Yunnan Pond frog *Dianrana pleuraden* [78] and the swelled vent frog *Feirana quadranus* [23]. The discriminative effect of sexual selection pressure on testis size remains unclear, due to the lack of population-specific OSR within each population, and further studies are needed.

Rainfall is thought to be associated with breeding phenology in most amphibians [83,84]. There is evidence that frogs inhabiting arid or semi-arid environments that initiate brief reproductive activity following rainfall events have shorter breeding periods and exhibit less inter-male competition [34]. In contrast, frogs that rely on continuous moisture to develop eggs may have prolonged breeding periods that coincide with seasonally occurring rainfall, exhibiting heightened inter-male competition [31]. Therefore, in general, rainfall influences the degree of inter-male competition and thereby promotes the local adaptation of male reproductive investment [85]. This local adaptation was verified in *Pseudophryne guentheri*, where the positive correlations between testis size and rainfall indicated that males from xeric populations exhibited reduced investment in testes size [31]. However, our findings did not provide evidence for possible adaptations of testis size to rainfall. We attribute the lack of a correlation between rainfall and testis size in *B. andrewsi* to other factors (e.g., sex selection pressure and/or genetic factors) concealing the effect of rainfall on male reproductive investment.

We found that the CV of temperature was correlated with testis size among populations. A study on fish considered seasonal temperature variation as a reliable predictor or indicator of conditions in stream ecosystems that influence individual fitness and life-history traits [86]. Therefore, strategies favoring energy allocation to reproductive tissues would be considered to be correlated with temperature variation [38,87]. An experimental study on the Terai tree frog *Polypedates teraiensis* found that lower temperature promotes testicular recrudescence under laboratory conditions, suggesting that temperature is involved in the regulation of seasonal breeding in this species [88]. Moreover, seasonality often influences development time, resource acquisition, and breeding activity [89,90,91]. The observed negative relationship between the CV of temperature and testis size of *B. andrewsi* was in accordance with our prediction that species inhabiting stable environments (smaller CV of temperature) would increase reproductive investment. One possible explanation is that individuals prefer to allocate more energy for reproductive investment in a small CV of temperature. By contrast, individuals prefer to allocate limited energy to survival and thus have smaller testes that can be more adapted to fluctuating temperature environments [92,93].

## 5. Conclusions

In summary, we found considerable geographical variation in testes size among 14 *B. andrewsi* populations and a positive correlation between body condition and relative testes size. We found that altitude and/or latitude do not affect testes size, which is inconsistent with our predictions. In addition, rainfall cannot cause positive effects on male reproductive investment. However, we found that the coefficient of variation of temperature shows effects on testes size, suggesting individuals prefer to allocate limited energy to survival and thus have smaller testes in fluctuating environments with larger a CV in temperature. Further work on the effects of environmental conditions on male reproductive investment variations should focus on more ejaculate traits and disentangle the more specific factors driving these variabilities of ejaculate traits.

## Figures and Tables

**Table 1:** Sample size, site locations, climate data, SVL, body mass, and testis size for each *B. andrewsi* population. Note: climate data were obtained and averaged from the website https://www.meteoblue.com (accessed on 17 August 2022) based on latitude, longitude, and altitude data of the sampling sites.

Study Site	Sample Size	Latitude (N)	Longitude (E)	Altitude (m)	Temperature (°C)		Precipitation (mm)		SVL (mm) ±SE	Body Mass (mg) ±SE	Testis Size (mg) ±SE
					AT	BT	AR	BR			
MX	19	31.67	103.84	1553	11.33	11.83	648	151	76.22 ± 5.9	51070 ± 11100	30.32 ± 14.4
JL	20	29.01	101.50	2902	9.21	9.17	865	245	67.42 ± 3.5	27930 ± 5330	23.09 ± 7.9
LD	20	29.90	102.21	1477	5.71	5.50	744	241	68.82 ± 5.6	27900 ± 6560	20.74 ± 6.1
TP	21	26.74	102.47	1916	14.54	15.83	1080	156	70.18 ± 5.5	31000 ± 6030	15.09 ± 5.0
PX	23	28.52	102.65	1864	13.58	13.83	1512	386	65.86 ± 8.2	29670 ± 12960	12.47 ± 8.4
AN	22	27.95	102.15	1522	15.92	16.67	594	144	67.87 ± 7.6	34380 ± 12550	19.31 ± 10.5
JC	25	31.30	102.04	2078	8.50	8.50	810	250	72.72 ± 3.3	37360 ± 6060	14.91 ± 5.1
HY	22	29.35	102.64	864	9.42	9.83	1491	423	62.41 ± 9.4	32540 ± 14220	22.71 ± 14.6
GC	26	26.83	100.28	2367	12.67	12.67	1492	242	79.22 ± 5.6	59120 ± 10720	22.85 ± 6.2
LX	28	31.42	103.21	2180	4.00	4.00	980	320	72.65 ± 3.3	38330 ± 4620	17.95 ± 6.6
DY	25	25.29	102.88	1995	14.83	12.75	894	175	64.42 ± 4.5	34360 ± 7870	18.94 ± 7.2
LG	30	28.29	102.19	1632	15.75	16.17	702	158	64.11 ± 5.4	26310 ± 7230	13.62 ± 4.2
ZJ	30	28.01	102.85	2050	12.63	13.67	1157	246	73.43 ± 5.0	40050 ± 9550	18.83 ± 8.2
XYB	20	33.56	108.52	1393	11.04	12.00	772	196	64.18 ± 4.6	29210 ± 4690	10.83 ± 2.1

AT: mean annual temperature; BT: temperature during the breeding season; AR: annual rainfall; BR: rainfall during the breeding season; MX: Maoxian; JL: Jiulong; LD: Luding; TP: Taiping; PX: Puxiong; AN: Anning; JC: Jinchuan; HY: Hanyuan; GC: Gucheng; LX: Lixian; DY: Dianyuan; LG: Lugu; ZJ: Zhaojue; XYB: Xunyangba.

**Table 2:** The variations of testis size in populations of Andrew’s toads (*B. andrewsi*) when correcting for body condition using ANCOVA.

Source	*Df*	Sum Sq	Mean Sq	*F*	*p*
Population	13	3.971	0.305	14.260	<0.001
Body condition	1	3.898	3.898	181.950	<0.001
Residuals	316	6.770	0.021		

**Table 3:** The influences of geographic gradients, climatic gradients, and population on variation in testis size across 14 populations of Andrew’s toads (*B. andrewsi*) when correcting for body condition using LMMs.

Source	Random		Fixed				
	VAR	SD	Estimate	SE	*df*	*t*	*P*
Geographic gradients							
Population	0.013	0.112					
Residual	0.021	0.147					
Latitude			−0.377	0.998	10.691	−0.378	0.713
Altitude			−0.072	0.266	10.771	−0.271	0.792
Body condition			2.298	0.169	322.526	13.564	<0.001
Climatic gradients							
Population	0.009	0.093					
Residual	0.021	0.146					
Mean annual temperature			−1.989	1.089	29.565	−1.827	0.078
Annual rainfall			−0.474	0.379	7.938	−1.251	0.246
CV of temperature			−0.959	0.359	11.865	−2.672	0.021
CV of rainfall			−0.038	0.077	7.632	−0.495	0.635
Temperature during breeding season			0.513	0.859	18.478	0.597	0.558
Rainfall during breeding season			0.155	0.400	7.908	0.387	0.709
Body condition			2.281	0.169	301.979	13.465	<0.001

**Table 4:** The influences of geographic gradients and climatic gradients on variation in mean testis size across 14 populations of Andrew’s toads (*B. andrewsi*) when correcting for body condition using GLMs.

Variable	*β*	SE	*t*	*P*
Geographic gradients				
Latitude	−0.413	0.990	−0.417	0.686
Altitude	−0.066	0.263	−0.251	0.807
Body condition	1.497	0.737	2.030	0.070
Climatic gradients				
Mean annual temperature	−2.069	1.941	−1.066	0.327
Annual rainfall	−0.391	0.485	−0.807	0.450
CV of temperature	−0.905	0.509	−1.780	0.125
CV of rainfall	−0.051	0.091	−0.565	0.593
Temperature during breeding season	0.677	1.414	0.479	0.649
Rainfall during breeding season	0.151	0.479	0.315	0.764
Body condition	1.964	0.836	2.349	0.057

## Data Availability

The data presented in this study are available on request from the corresponding author. The data are not publicly available due to privacy or ethical restrictions.

## References

[1] Lack D. (1966). Population Studies on Birds.

[2] Stearns S.C. (1989). The evolutionary significance of phenotypic plasticity. Bioscience.

[3] Hettyey A., Laurila A., Herczeg G., Jonsson K., Kovacs T., Merila J. (2005). Does testis weight decline towards the Subarctic? A case study on the common frog, *Rana temporaria*. Naturwissenschaften.

[4] Balciauskas L., Amshokova A., Balciauskiene L., Benedek A.M., Cichocki J., Csanady A., De Mendonca P.G., Nistreanu V. (2020). Geographical clines in the size of the herb field mouse (*Apodemus uralensis*). Integr. Zool..

[5] Clifton I.T., Chamberlain J.D., Gifford M.E. (2020). Role of phenotypic plasticity in morphological differentiation between water snake populations. Integr. Zool..

[6] Imakando C.I., Fernandez-Grandon G.M., Singleton G.R., Belmain S.R. (2021). Impact of fertility versus mortality control on the demographics of *Mastomys natalensis* in maize fields. Integr. Zool..

[7] Peng Z.W., Zhang L.X., Lu X. (2022). Global gaps in age data based on skeletochronology for amphibians. Integr. Zool..

[8] Pitcher T.E., Stutchbury B.J.M. (1998). Latitudinal variation in testis size in six species of North American songbirds. Can. J. Zool..

[9] Dziminski M.A., Roberts J.D., Beveridge M., Simmons L.W. (2010). Among-population covariation between sperm competition and ejaculate expenditure in frogs. Behav. Ecol..

[10] Zeng Y., Lou S.L., Liao W.B., Jehle R. (2014). Evolution of sperm morphology in anurans: Insights into the roles of mating system and spawning location. BMC Evol. Biol..

[11] Olarte O., Sanchez-Montes G., Martinez-Solano I. (2020). Integrative demographic study of the Iberian painted frog (*Discoglossus galganoi*): Inter-annual variation in the effective to census population size ratio, with insights on mating system and breeding success. Integr. Zool..

[12] Liao W.B., Mi Z.P., Li C.L., Wei S.C., Wu H. (2013). Sperm traits in relation to male amplexus position in the Omei treefrog *Rhacophorus omeimontis*, a species with group spawning. J. Herpetol..

[13] Liao W.B., Xiao W.M., Cai Y.L. (2013). Within population variation in testis size in the mole-shrew (*Anourosorex squamipes*). Ital. J. Zool..

[14] Chen C., Huang Y.Y., Liao W.B. (2016). A comparison of testes size and sperm length between *Polypedates megacephalus* populations at different altitudes. Herpetol. J..

[15] Parker G.A., Birkhead T.R., Møller A.P. (1998). Sperm competition and the evolution of ejaculates: Towards a theory base. Sperm Competition and Sexual Selection.

[16] Parker G.A. (2016). The evolution of expenditure on testes. J. Zool..

[17] Lüpold S., de Boer R.A., Evans J.P., Tomkins J.L., Fitzpatrick J.L. (2020). How sperm competition shapes the evolution of testes and sperm: A meta-analysis. Phil. Trans. R. Soc. B.

[18] Emlen S.T., Oring L.W. (1977). Ecology, sexual selection, and the evolution of mating systems. Science.

[19] Kvarnemo C., Ahnesjo I. (1996). The dynamics of operational sex ratios and competition for mates. Trends Ecol. Evol..

[20] Laiolo P., Illera J.C., Obeso J.R. (2013). Local climate determines intra- and interspecific variation in sexual size dimorphism in mountain grasshopper communities. J. Evol. Biol..

[21] Jin L., Mi Z.P., Liao W.B. (2016). Altitudinal variation in male reproductive investment in a polyandrous frog species (*Hyla gongshanensis jingdongensis*). Anim. Biol..

[22] Jin L., Yang S.N., Liao W.B., Lüpold S. (2016). Altitude underlies variation in the mating system, somatic condition, and investment in reproductive traits in male Asian grass frogs (*Fejervarya limnocharis*). Behav. Ecol. Sociobiol..

[23] Tang T., Luo Y., Huang C.H., Liao W.B., Huang W.C. (2018). Variation in somatic condition and testis mass in *Feirana quadranus* along an altitudinal gradient. Anim. Biol..

[24] Donihue C.M., Daltry J.C., Challenger S., Herrel A. (2021). Population increase and changes in behavior and morphology in the Critically Endangered Redonda ground lizard (*Pholidoscelis atratus*) following the successful removal of alien rats and goats. Integr. Zool..

[25] Liu Y.T., Wu Z.J., Liao W.B. (2022). Large-brained birds display lower extra-pair paternity. Integr. Zool..

[26] Briskie J.V. (1992). Copulation patterns and sperm competition in the polygynandrous Smith’s longspur. Auk.

[27] Stutchbury B.J., Morton E.S. (1995). The effect of breeding synchrony on extra-pair mating systems in songbirds. Behaviour.

[28] Morrison C., Hero J.M. (2003). Geographic variation in life-history characteristics of amphibians: A review. J. Anim. Ecol..

[29] Wingfield J.C. (2008). Organization of vertebrate annual cycles: Implications for control mechanisms. Phil. Trans. R. Soc. B.

[30] Jiang A., Zhong M.J., Xie M., Lou S.L., Jin L., Robert J., Liao W.B. (2015). Seasonality and age is positively related to brain size in Andrew’s toad (*Bufo andrewsi*). Evol. Biol..

[31] Rudin-Bitterli T.S., Mitchell N.J., Evans J.P. (2020). Extensive geographical variation in testes size and ejaculate traits in a terrestrial-breeding frog. Biol. Lett..

[32] Yu W.Y., Fan S.J., Wang X., Zhu J.Y., Yuan Z.R., Han Y.Y., Zhang H.L., Weng Q. (2022). Seasonal change of circulating leptin associated with testicular activities of the wild ground squirrels (*Citellus dauricus*). Integr. Zool..

[33] Evans J.P., Garcia-Gonzalez F. (2016). The total opportunity for sexual selection and the integration of pre- and post-mating episodes of sexual selection in a complex world. J. Evol. Biol..

[34] Lüpold S., Jin L., Liao W.B. (2017). Population density and structure drive differential investment in pre- and postmating sexual traits in frogs. Evolution.

[35] Liang T., Meiri S., Shi L. (2022). Sexual size dimorphism in lizards: Rensch’s rule, reproductive mode, clutch size, and line fitting method effects. Integr. Zool..

[36] Espunyes J., Serrano E., Chaves S., Bartolomé J., Menaut P., Albanell E., Marchand P., Foulché K., Garel M. (2022). Positive effect of spring advance on the diet quality of an alpine herbivore. Integr. Zool..

[37] Snook R.R. (2001). Absence of latitudinal clines in sperm characters in North American populations of *Drosophila subobscura* (Diptera: Drosophilidae). Pan-Pac. Entomol..

[38] Blanckenhorn W., Hellriegel B. (2002). Against Bergmann’s rule: Fly sperm size increases with temperature. Ecol. Lett..

[39] Lüpold S., Westneat D.F., Birkhead T.R. (2011). Geographical variation in sperm morphology in the red-winged blackbird (*Agelaius phoeniceus*). Evol. Ecol..

[40] Chen W., Pike D.A., He D.J., Wang Y., Ren L.N., Wang X.Y., Fan X.G., Lu X. (2014). Altitude decreases testis weight of a frog (*Rana kukunoris*) on the Tibetan plateau. Herpetol. J..

[41] Selemani M., Makundi R.H., Massawe A.W., Mhamphi G., Mulungu L.S., Belmain S.R. (2021). Impact of contraceptive hormones on the reproductive potential of male and female commensal black rats (*Rattus rattus*). Integr. Zool..

[42] Tranquillo C., Wauters L.A., Santicchia F., Preatoni D., Martinoli A. (2022). Living on the edge: Morphological and behavioral adaptations to a marginal high-elevation habitat in an arboreal mammal. Integr. Zool..

[43] Hettyey A., Roberts J.D. (2006). Sperm traits of the quacking frog, *Crinia georgiana*: Intra-and interpopulation variation in a species with a high risk of sperm competition. Behav. Ecol. Sociobiol..

[44] Zhang L., An D.C., He Y., Li Z., Fang B., Chen X., Lu X. (2018). Variation in testis weight of the Tibetan toad *Scutiger boulengeri* along a narrow altitudinal gradient. Anim. Biol..

[45] Fei L., Ye C.Y. (2001). The Colour Handbook of Amphibians of Sichuan.

[46] Zhu X., Chen C., Jiang Y., Zhao L., Jin L. (2022). Geographical variation of organ size in Andrew’s toad (*Bufo andrewsi*). Front. Ecol. Evol..

[47] Liao W.B., Lu X. (2012). Adult body size = f (initial size + growth rate × age): Explaining the proximate cause of Bergman’s cline in a toad along altitudinal gradients. Evol. Ecol..

[48] Liao W.B., Liu W.C., Merilä J. (2015). Andrew meets Rensch: Sexual size dimorphism and the inverse of Rensch’s rule in Andrew’s toad (*Bufo andrewsi*). Oecologia.

[49] Liao W.B., Lou S.L., Zeng Y., Kotrschal A. (2016). Large brains, small guts: The expensive tissue hypothesis supported in anurans. Am. Nat..

[50] Yang S.N., Huang X.F., Zhong M.J., Liao W.B. (2017). Geographical variation in limb muscle mass of the Andrew’s toad (*Bufo andrewsi*). Anim. Biol..

[51] Zhao L., Mai C.L., Liu G.H., Liao W.B. (2019). Altitudinal implications in organ size in the Andrew’s toad (*Bufo andrewsi*). Anim. Biol..

[52] Guo B.C., Lu D., Liao W.B., Merilä J. (2016). Genomewide scan for adaptive differentiation along altitudinal gradient in the Andrew's toad *Bufo andrewsi*. Mol. Ecol..

[53] Jin L., Liu W.C., Li Y.H., Zeng Y., Liao W.B. (2015). Evidence for the expensive-tissue hypothesis in the Omei wood frog (*Rana omeimontis*). Herpetol. J..

[54] Yu X., Zhong M.J., Li D.Y., Jin L., Liao W.B., Kotrschal A. (2018). Large–brained frogs mature later and live longer. Evolution.

[55] Jiang Y., Chen C., Liao W.B. (2022). Anuran interorbital distance variation: The role of ecological and behavioral factors. Integr. Zool..

[56] Zeng Y., Lou S.L., Liao W.B., Jehle R., Kotrschal A. (2016). Sexual selection impacts brain anatomy in frogs and toads. Ecol. Evol..

[57] Gu J., Li D.Y., Luo Y., Ying S.B., Zhang L.Y., Shi Q.M., Chen J., Zhang S.P., Zhou Z.M., Liao W.B. (2017). Brain size in *Hylarana guentheri* seems unaffected by variation in temperature and growth season. Anim. Biol..

[58] Zhong M.J., Wang X.Y., Huang Y.Y., Liao W.B. (2017). Altitudinal variation in organ size in *Polypedates megacephalus*. Herpetol. J..

[59] The R Project for Statistical Computing. https://www.R-project.org/.

[60] Mi Z.P., Liao W.B., Jin L., Lou S.L., Cheng J., Wu H. (2012). Testis asymmetry and sperm length in *Rhacophorus omeimontis*. Zool. Sci..

[61] Bates D., Mächler M., Bolker B., Walker S. (2015). Fitting linear mixed-effects models using lme4. J. Stat. Soft..

[62] Luo Y., Zhong M.J., Huang Y., Li F., Liao W.B., Kotrschal A. (2017). Seasonality and brain size are negatively associated in frogs: Evidence for the expensive brain framework. Sci. Rep..

[63] Harcourt A.H., Harvey P.H., Larson S.G., Short R.V. (1981). Testis weight, body weight and breeding system in primates. Nature.

[64] Jamieson B.G.M., Briskie J.V., Montgomerie R., Jamieson B.G.M. (2007). Testis size, sperm size and sperm competition. Reproductive Biology and Phylogeny of Birds. Part A: Phylogeny, Morphology, Hormones, Fertilization.

[65] Emerson S.B. (1997). Testis size variation in frogs: Testing the alternatives. Behav. Ecol. Sociobiol..

[66] Byrne P., Roberts J.D., Simmons L.W. (2002). Sperm competition selects for increased testis mass in Australian frogs. J. Evol. Biol..

[67] Møller A.P. (1994). Directional selection on directional asymmetry: Testes size and secondary sexual characters in birds. Proc. R Soc. Lond. B.

[68] Simmons L.W., Kotiaho J.S. (2002). Evolution of ejaculates: Patterns of phenotypic and genotypic variation and condition dependence in sperm competition traits. Evolution.

[69] Schulte-Hostedde A.I., Millar J.S. (2004). Intraspecific variation of testis size and sperm length in the yellow-pine chipmunk (*Tamias amoenus*): Implications for sperm competition and reproductive success. Behav. Ecol. Sociobiol..

[70] Schulte-Hostedde A.I., Millar J.S., Hickling G.J. (2005). Condition dependence of testis size in small mammals. Evol. Ecol. Res..

[71] Burness G., Schulte-Hostedde A.I., Montgomerie R. (2008). Body condition influences sperm energetics in lake whitefish (*Coregonus clupeaformis*). Can. J. Fish Aquat. Sci..

[72] Liu Y.H., Liao W.B., Zhou C.Q., Mi Z.P., Mao M. (2011). Asymmetry of testes in Guenther’s frog, *Hylarana guentheri* (Anuar: Ranidae). Asian Herpetol. Res..

[73] Zhou C.Q., Mao M., Liao W.B., Mi Z.P., Liu Y.H. (2011). Testis asymmetry in the dark-spotted frog *Rana nigromaculata*. Herpetol. J..

[74] Dewsbury D.A. (1982). Ejaculate cost and male choice. Am. Nat..

[75] Olsson M., Madsen T., Shine R. (1997). Is sperm really so cheap? Costs of reproduction in male adders, *Vipera berus*. Proc. R Soc. Lond. B.

[76] Thomsen R., Soltis J., Matsubara M., Matsubayashi K., Onuma M., Takenaka O. (2006). How costly are ejaculates for Japanese macaques?. Primates.

[77] Preston B.T., Stevenson I.R., Pemberton J.M., Coltman D.W., Wilson K. (2003). Overt and covert competition in a promiscuous mammal: The importance of weaponry and testes size to male reproductive success. Proc. R Soc. Lond. B.

[78] Mai C.L., Liu Y.H., Jin L., Mi Z.P., Liao W.B. (2017). Altitudinal variation in somatic condition and investment in reproductive traits in male Yunnan Pond frog (*Dianrana pleuraden*). Zool. Anz..

[79] Zhao L., Mao M., Liao W.B. (2016). No evidence for the ‘expensive-tissue hypothesis’ in the dark-spotted frog (*Pelophylax nigromaculata*). Acta Herpetol..

[80] Wu Q.G., Liao W.B. (2017). Evidence for directional testes asymmetry in *Hyla gongshanensis jindongensis*. Acta Herpetol..

[81] Yue Y.F., Jin L., Mai C.L., Huang X.F., Liao W.B. (2020). No evidence for the compensation hypothesis in the swelled vent frog (*Feirana quadranus*). Asian Herpetol. Res..

[82] Liao W.B., Lu X. (2010). Breeding behavior of the Omei treefrog *Rhacophorus omeimontis* (Anura: Rachophoridae) in a subtropical montane region. J. Nat. Hist..

[83] Corn P.S. (2003). Amphibian breeding and climate change: Importance of snow in the mountains. Conserv. Biol..

[84] Walls S., Barichivich W., Brown M. (2013). Drought, deluge and declines: The impact of precipitation extremes on amphibians in a changing climate. Biology.

[85] Álvarez D., Viesca L., Nicieza A. (2014). Sperm competitiveness differs between two frog populations with different breeding systems. J. Zool..

[86] Beschta R.L., Bilby R.E., Brown G.W., Holtby L.B., Hofstra T.D., Salo E.O., Cundy T.W. (1989). Stream temperature and aquatic habitat: Fisheries and forestry interactions. Streamside Management: Forestry and Fishery Interactions.

[87] Souza U.P., Ferreira F.C., Braga F.M.D.S., Winemiller K.O. (2015). Feeding, body condition and reproductive investment of *Astyanax intermedius* (Characiformes, Characidae) in relation to rainfall and temperature in a Brazilian Atlantic Forest stream. Ecol. Freshw. Fish.

[88] Borah B.K., Renthlei Z., Trivedi A.K. (2019). Seasonality in terai tree frog (*Polypedates teraiensis*): Role of light and temperature in regulation of seasonal breeding. J. Photochem. Photobiol. B.

[89] Hjernquist M.B., Söderman F., Jönsson K.I., Herczeg G., Laurila A., Merilä J. (2012). Seasonality determines patterns of growth and age structure over a geographic gradient in an ectothermic vertebrate. Oecologia.

[90] Liao W.B., Luo Y., Lou S.L., Jehle R. (2016). Geographic variation in life-history traits: Growth season affects age structure, egg size and clutch size in Andrew’s toad (*Bufo andrewsi*). Front. Zool..

[91] Alton L.A., Condon C., White C.R., Angilletta M.J. (2017). Jr Colder environments did not select for a faster metabolism during experimental evolution of *Drosophila melanogaster*. Evolution.

[92] Valenzuela-Sanchez A., Cunningham A.A., Soto-Azat C. (2015). Geographic body size variation in ectotherms: Effects of seasonality on an anuran from the southern temperate forest. Front. Zool..

[93] Fu L., Wang X., Yang S., Li C., Hu J. (2022). Morphological variation and its environmental correlates in the Taihangshan swelled-vented frog across the Qinling mountains. Animals.

